# Complete genome sequence of *Intrasporangium calvum* type strain (7 KIP^T^)

**DOI:** 10.4056/sigs.1263355

**Published:** 2010-11-16

**Authors:** Tijana Glavina Del Rio, Olga Chertkov, Montri Yasawong, Susan Lucas, Shweta Deshpande, Jan-Fang Cheng, Chris Detter, Roxanne Tapia, Cliff Han, Lynne Goodwin, Sam Pitluck, Konstantinos Liolios, Natalia Ivanova, Konstantinos Mavromatis, Amrita Pati, Amy Chen, Krishna Palaniappan, Miriam Land, Loren Hauser, Yun-Juan Chang, Cynthia D. Jeffries, Manfred Rohde, Rüdiger Pukall, Johannes Sikorski, Markus Göker, Tanja Woyke, James Bristow, Jonathan A. Eisen, Victor Markowitz, Philip Hugenholtz, Nikos C. Kyrpides, Hans-Peter Klenk, Alla Lapidus

**Affiliations:** 1 DOE Joint Genome Institute, Walnut Creek, California, USA; 2 Los Alamos National Laboratory, Bioscience Division, Los Alamos, New Mexico, USA; 3 HZI – Helmholtz Centre for Infection Research, Braunschweig, Germany; 4 Biological Data Management and Technology Center, Lawrence Berkeley National Laboratory, Berkeley, California, USA; 5 Oak Ridge National Laboratory, Oak Ridge, Tennessee, USA; 6 DSMZ - German Collection of Microorganisms and Cell Cultures GmbH, Braunschweig, Germany; 7 University of California Davis Genome Center, Davis, California, USA

**Keywords:** airborne, Gram-positive, non-motile, intercalary vesicles, nocardioform, *Actinobacteria*, *Intrasporangiaceae*, GEBA

## Abstract

*Intrasporangium calvum* Kalakoutskii *et al.* 1967 is the type species of the genus *Intrasporangium*, which belongs to the actinobacterial family *Intrasporangiaceae*. The species is a Gram-positive bacterium that forms a branching mycelium, which tends to break into irregular fragments. The mycelium of this strain may bear intercalary vesicles but does not contain spores. The strain described in this study is an airborne organism that was isolated from a school dining room in 1967. One particularly interesting feature of *I. calvum* is that the type of its menaquinone is different from all other representatives of the family *Intrasporangiaceae*. This is the first completed genome sequence from a member of the genus *Intrasporangium* and also the first sequence from the family *Intrasporangiaceae*. The 4,024,382 bp long genome with its 3,653 protein-coding and 57 RNA genes is a part of the *** G****enomic* *** E****ncyclopedia of* *** B****acteria and* *** A****rchaea * project.

## Introduction

Strain 7 KIP^T^ (= DSM 43043 = ATCC 23552 = JCM 3097) is the type strain of the species *Intrasporangium calvum*, which is the type species of its genus *Intrasporangium* [1,2]. The generic name derived from the Latin word *intra* meaning *within* and the Greek word *spora* meaning *a seed*. The name *Intrasporangium*, was selected to emphasize the possibility of intercalary formation of sporangia in mycelial filaments [3]. *Intrasporangium* is the type genus of the family *Intrasporangiaceae* and one out of currently nineteen genera in the family *Intrasporangiaceae* [4-6]. Strain 7 KIP^T^ was first described in 1967 by Kalakoutskii *et al.* as an airborne organism, which was isolated under nonselective conditions on plates of meat-peptone agar exposed to the atmosphere of a school dining room [1,7,8]. *I. calvum* is of particular interest because the type of its menaquinones is different from all other representatives of the family *Intrasporangiaceae* [8]. Here we present a summary classification and a set of features for *I. calvum* 7 KIP^T^, together with the description of the complete genomic sequencing and annotation.

## Classification and features

The 16S rRNA gene of strain 7 KIP^T^ shares 92.6-98.7% sequence identity with the sequences of the type strains from the other members of the family *Intrasporangiaceae* [9], with *Humihabitans oryzae* as the closest relative. The 16S rRNA gene sequence of 7 KIP^T^ is 99% identical to the uncultured *Intrasporangiaceae* clone HT06Ba24, isolated from soil of a former coal gasification site in Gliwice, Poland [10,11] and AKAU4164, isolated from uranium contaminated soil in Oak Ridge, USA [10,12]. The environmental samples database (env_nt) contains the marine metagenome clone 1096626841081 (AACY020552144) from surface water (92% sequence identity with 7 KIP^T^). The genomic survey sequences database (gss) contains the metagenomic clone 1061002660518 from Floreana island in Punta Cormorant, Ecuador [10], which shares 93% sequence identity with 7 KIP^T^ (as of July 2010). One of the 16S rRNA sequences of strain 7 KIP^T^ was compared using NCBI BLAST under default values (e.g., considering only the best 250 hits) with the most recent release of the Greengenes database [13] and the relative frequencies, weighted by BLAST scores, of taxa and keywords, weighted by BLAST scores, were determined. The five most frequent genera were *Janibacter* (29.6%), *Terrabacter* (19.8%), *Sanguibacter* (8.4%), *Dermacoccus* (7.7%) and *Tetrasphaera* (6.2%). The five most frequent keywords within the labels of environmental samples which yielded hits were 'skin' (9.1%), 'human' (4.7%), 'microbiome/temporal/topographical' (4.5%), 'sludge' (4.4%) and 'heel/plantar' (3.1%). The single most frequent keyword within the labels of environmental samples which yielded hits of a higher score than the highest scoring species was 'contaminated/soil/uranium' (33.3%).

**Figure 1:**
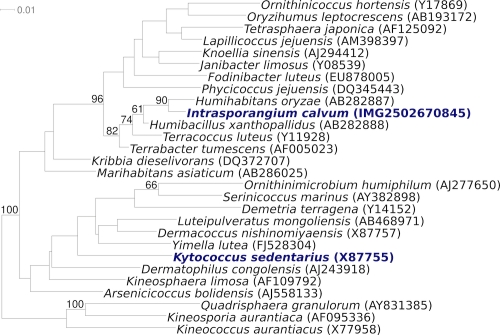
Phylogenetic tree highlighting the position of *I. calvum* 7 KIP^T^ relative to the type strains of the genera within the family *Intrasporangiaceae*. The trees were inferred from 1,406 aligned characters [14,15] of the 16S rRNA gene sequence under the maximum likelihood criterion [16] and rooted with the type strains of the genera within the family *Kineosporiaceae* [17]. The branches are scaled in terms of the expected number of substitutions per site. Numbers above branches are support values from 650 bootstrap replicates [18] if larger than 60%. Lineages with type strain genome sequencing projects registered in GOLD [19] are shown in blue, published genomes in bold [20].

Figure 1 shows the phylogenetic neighborhood of *I. calvum* 7 KIP^T^ in a 16S rRNA based tree. The sequences of the two 16S rRNA gene copies in the genome are differ by only one nucleotide from each other and by up to one nucleotide from the previously published sequence generated from DSM 43043 (AJ566282).

**Figure 2:**
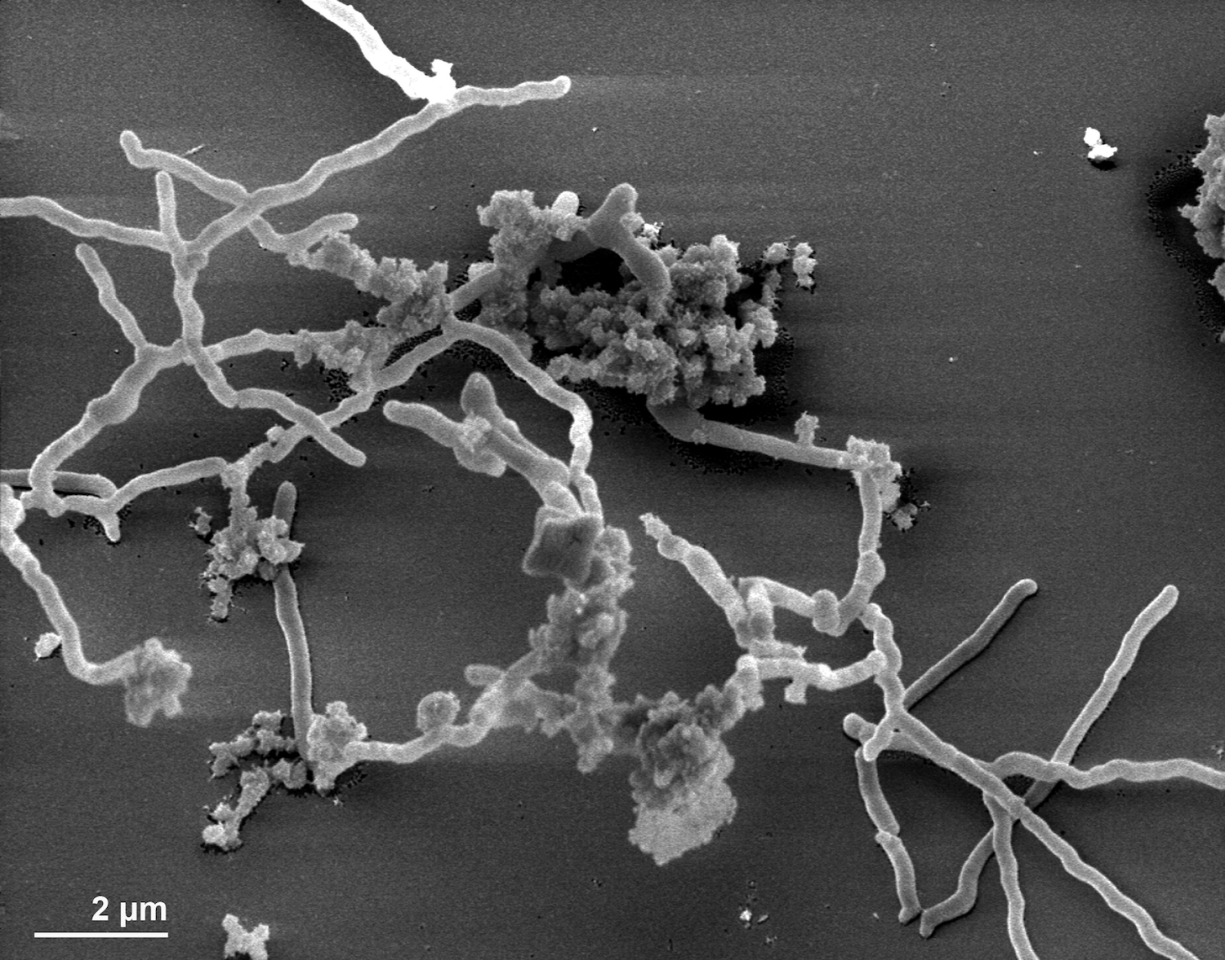
Scanning electron micrograph of *I. calvum* 7 KIP^T^

Strain 7 KIP^T^ forms a branching mycelium, which tends to break into irregular fragments, *i.e*., typically nocardioform [1,8]. The mycelium may bear intercalary vesicles that do not contain spores [Table 1, Figure 2, 7,26]. The vesicles of strain 7 KIP^T^ are ovoid and lemon-shaped (5-15 µm in diameter) [1,7]. Several round or oval bodies (1.2-1.5 µm in diameter) may be observed in the vesicles of older cultures [1,7]. The oval bodies in the vesicles of strain 7 KIP^T^ are nonmotile but may undergo a Brownian movement (in mature vesicles) [1,7]. There was no aerial mycelium observed from the strain 7 KIP^T^ [1,7,8]. The mycelial filaments penetrate the agar and form compact, small colonies (1-5 mm of diameter) [1]. These colonies are round, glistening and whitish (cream-whitish in old colonies) when the cells are grown on meat-extract peptone agar [1]. Strain 7 KIP^T^ is aerobic and Gram-positive (Gram-variable in old cultures) and not acid-fast [1]. Strain 7 KIP^T^ is rather fastidious in nutritional requirements [1]. Growth is seemingly dependent on some unidentified substances present in the peptone used in the growth medium [1]. The strain prefers complex media for growth, especially containing peptone and yeast extract [1,7]. As such, the growth characteristics on a variety of media such as meat-extract peptone, blood serum broth, oatmeal agar, Sauton medium agar and other media, also in combination of different atmospheric gases and their concentrations, have been studied in detail [1]. Strain 7 KIP^T^ is able to grow between 28°C and 37°C, however, the cells grow faster at 37°C than 28°C, but it does not at 45°C [1]. It grows slowly on meat-extract peptone medium and the first signs of macroscopic growth will appear after 3-5 days when incubated at 28°C [1]. Strain 7 KIP^T^ does not grow on the majority of synthetic mineral media that are routinely used for actinomycetes [1,7]. Strain 7 KIP^T^ is able to reduce nitrate to nitrite when KNO_3_ is added to the growth medium (meat-extract peptone broth) [1]. The liquefaction of gelatin does not occur when the strain 7 KIP^T^ was grown on meat-extract peptone gelatine [1]. Strain 7 KIP^T^ has no antibiotic activity against *Micrococcus luteus*, *Staphylococcus aureus*, *Escherichia coli*, *Bacillus subtilis*, *Candida albicans* and *Mycobacterium* sp. v-5 [1]

**Table 1:** Classification and general features of *I. calvum* 7 KIP^T^ according to the MIGS recommendations [21].

**MIGS ID**	**Property**	**Term**	**Evidence code**
	Current classification	Domain *Bacteria*	TAS [22]
		Phylum *Actinobacteria*	TAS [23]
		Class *Actinobacteria*	TAS [4,24]
		Subclass *Actinobacteridae*	TAS [4,6]
		Order *Actinomycetales*	TAS [2,4,6,25]
		Suborder *Micrococcineae*	TAS [4,6]
		Family *Intrasporangiaceae*	TAS [4-6]
		Genus *Intrasporangium*	TAS [1,2]
		Species *Intrasporangium calvum*	TAS [1,2]
		Type strain 7 KIP	TAS [1]
	Gram stain	positive	TAS [1]
	Cell shape	branching mycelium, which tends to break into irregular fragments	TAS [1,8]
	Motility	none	TAS [1,26]
	Sporulation	none	TAS [26]
	Temperature range	28°C–37°C	TAS [1]
	Optimum temperature	37°C	NAS
	Salinity	not reported	
MIGS-22	Oxygen requirement	aerobic	TAS [1,7]
	Carbon source	carbohydrates	TAS [1]
	Energy source	chemoorganotroph	TAS [1,7]
MIGS-6	Habitat	air	TAS [1]
MIGS-15	Biotic relationship	free-living	NAS
MIGS-14	Pathogenicity	none	NAS
	Biosafety level	1	TAS [27]
	Isolation	air in a school dining room	TAS [1]
MIGS-4	Geographic location	Russia	NAS
MIGS-5	Sample collection time	1967	TAS [1]
MIGS-4.1	Latitude	not reported	
MIGS-4.2	Longitude	not reported	
MIGS-4.3	Depth	not reported	
MIGS-4.4	Altitude	not reported	

Evidence codes - IDA: Inferred from Direct Assay (first time in publication); TAS: Traceable Author Statement (i.e., a direct report exists in the literature); NAS: Non-traceable Author Statement (i.e., not directly observed for the living, isolated sample, but based on a generally accepted property for the species, or anecdotal evidence). These evidence codes are from of the Gene Ontology project [28]. If the evidence code is IDA, then the property was directly observed by one of the authors or an expert mentioned in the acknowledgements.

### Chemotaxonomy

Strain 7 KIP^T^ contains LL-diaminopimelic acid (LL-A_2_pm) in the cell wall and possesses the A3γ-type of peptidoglycan [29,30]. The amino acid at position 1 of the peptide subunit is L-alanine [30]. The cell wall structure of strain 7 KIP^T^ is characterized by the cross-linkage of the A3γ-type peptidoglycan via a triglycine-interpeptide bridge and by a glycine residue bound to the α-carboxyl group of the D-glutamic acid position 2 of the peptide subunit [29,30]. Strain 7 KIP^T^ possesses a totally unsaturated menaquinone with eight isoprene units (MK-8) instead of a partially saturated menaquinone with two of eight isoprene units hydrogenated (MK-8(H_4_)) which is the characteristic menaquinone of all other representatives of the family *Intrasporangiaceae* [8,30]. Cells of strain 7 KIP^T^ contain glucosamine-containing phospholipids (phospholipids type 4) [7]. Polar lipids of the strain are phosphatidyl-inositol, phosphatidylinositol mannosides, phosphatidylglycerol and diphosphatidyl-glycerol [30]. The major cellular fatty acids are saturated branched-chain acids: iso-C_15:0_ (37.8%), anteiso-C_15:0_ (12.6%), iso-C_16:0_ (12.3%), iso-C_14:0_ (5.0%), anteiso-C_17:0_ (3.9%), iso-C_17:1_ (3.7%), iso-C_15:1_ (3.5%), iso-C_16:1_ (3.1%) and straight chain acid C_15:0_ (2.7%) [30]. Polyamine contents (µmol per g dry wt) of strain 7 KIP^T^ are putrescine (2.02), spermidine (1.03), spermine (0.31), cadaverine (0.30), 1,3-diaminopropane (0.17), *sym*-homospermidine (0.05) and tyramine (0.17) [29].

## Genome sequencing and annotation

### Genome project history

This organism was selected for sequencing on the basis of its phylogenetic position [31], and is part of the *** G****enomic* *** E****ncyclopedia of* *** B****acteria and* *** A****rchaea * project [32]. The genome project is deposited in the Genome OnLine Database [19] and the complete genome sequence is deposited in GenBank. Sequencing, finishing and annotation were performed by the DOE Joint Genome Institute (JGI). A summary of the project information is shown in Table 2.

**Table 2:** Genome sequencing project information

**MIGS ID**	**Property**	**Term**
MIGS-31	Finishing quality	Finished
MIGS-28	Libraries used	Three genomic libraries: one standard and one paired ended 454 pyrosequence library and one standard Illumina library
MIGS-29	Sequencing platforms	454 GS FLX Titanium, Illumina GAii
MIGS-31.2	Sequencing coverage	59.7 × pyrosequence: 95.2 × Illumina
MIGS-30	Assemblers	Newbler version 2.0.0-PostRelease- 11/04/2008, phrap
MIGS-32	Gene calling method	Prodigal 1.4, GenePRIMP
	INSDC ID	CP002343
	Genbank Date of Release	December 29, 2010
	GOLD ID	Gc01572
	NCBI project ID	43527
	Database: IMG-GEBA	2503538011
MIGS-13	Source material identifier	DSM 43043
	Project relevance	Tree of Life, GEBA

### Growth conditions and DNA isolation

*I. calvum* 7 KIP^T^ was grown in medium 65 (GYM Streptomycetes medium) supplemented with one third of BHI (medium 215) [33] at 28°C. DNA was isolated from 0.5-1 g of cell paste using Qiagen Genomic 500 DNA Kit (Qiagen, Hilden, Germany) following the standard protocol as recommended by the manufacturer, with modification st/LALMP for cell lysis as described by Wu *et al.* [32].

### Genome sequencing and assembly

The genome was sequenced using a combination of Illumina and 454 sequencing platforms. All general aspects of library construction and sequencing can be found at the JGI website [34]. Pyrosequencing reads were assembled using the Newbler assembler version 2.0.0-PostRelease-11/04/2008 (Roche). The initial Newbler assembly consisted of 28 contigs in two scaffolds and was converted into a phrap assembly by making fake reads from the consensus, collecting the read pairs in the 454 paired end library. Illumina GAii sequencing data (309MB) was assembled with Velvet [35] and the consensus sequences were shredded into 1.5 kb overlapped fake reads and assembled together with the 454 data. The 454 draft assembly was based on 226.2 Mb 454 draft data and all of the 454 paired end data. Newbler parameters are -consed -a 50 -l 350 -g -m -ml 20. The Phred/Phrap/Consed software package [36] was used for sequence assembly and quality assessment in the following finishing process. After the shotgun stage, reads were assembled with parallel phrap (High Performance Software, LLC). Possible mis-assemblies were corrected with gapResolution [34], Dupfinisher, or sequencing cloned bridging PCR fragments with subcloning or transposon bombing (Epicentre Biotechnologies, Madison, WI) [20]. Gaps between contigs were closed by editing in Consed, by PCR and by Bubble PCR primer walks (J.-F.Chang, unpublished). A total of 139 additional reactions were necessary to close gaps and to raise the quality of the finished sequence. Illumina reads were also used to correct potential base errors and increase consensus quality using a software Polisher developed at JGI [37]. The error rate of the completed genome sequence is less than one error in 100,000. Together, the combination of the Illumina and 454 sequencing platforms provided 154.9 × coverage of the genome. The final assembly contains 847,906 pyrosequencing and 11,758,818 Illumina reads.

### Genome annotation

Genes were identified using Prodigal [38] as part of the Oak Ridge National Laboratory genome annotation pipeline, followed by a round of manual curation using the JGI GenePRIMP pipeline [39]. The predicted CDSs were translated and used to search the National Center for Biotechnology Information (NCBI) nonredundant database, UniProt, TIGRFam, Pfam, PRIAM, KEGG, COG, and InterPro databases. Additional gene prediction analysis and functional annotation was performed within the Integrated Microbial Genomes - Expert Review (IMG-ER) platform [40].

## Genome properties

**Figure 3:**
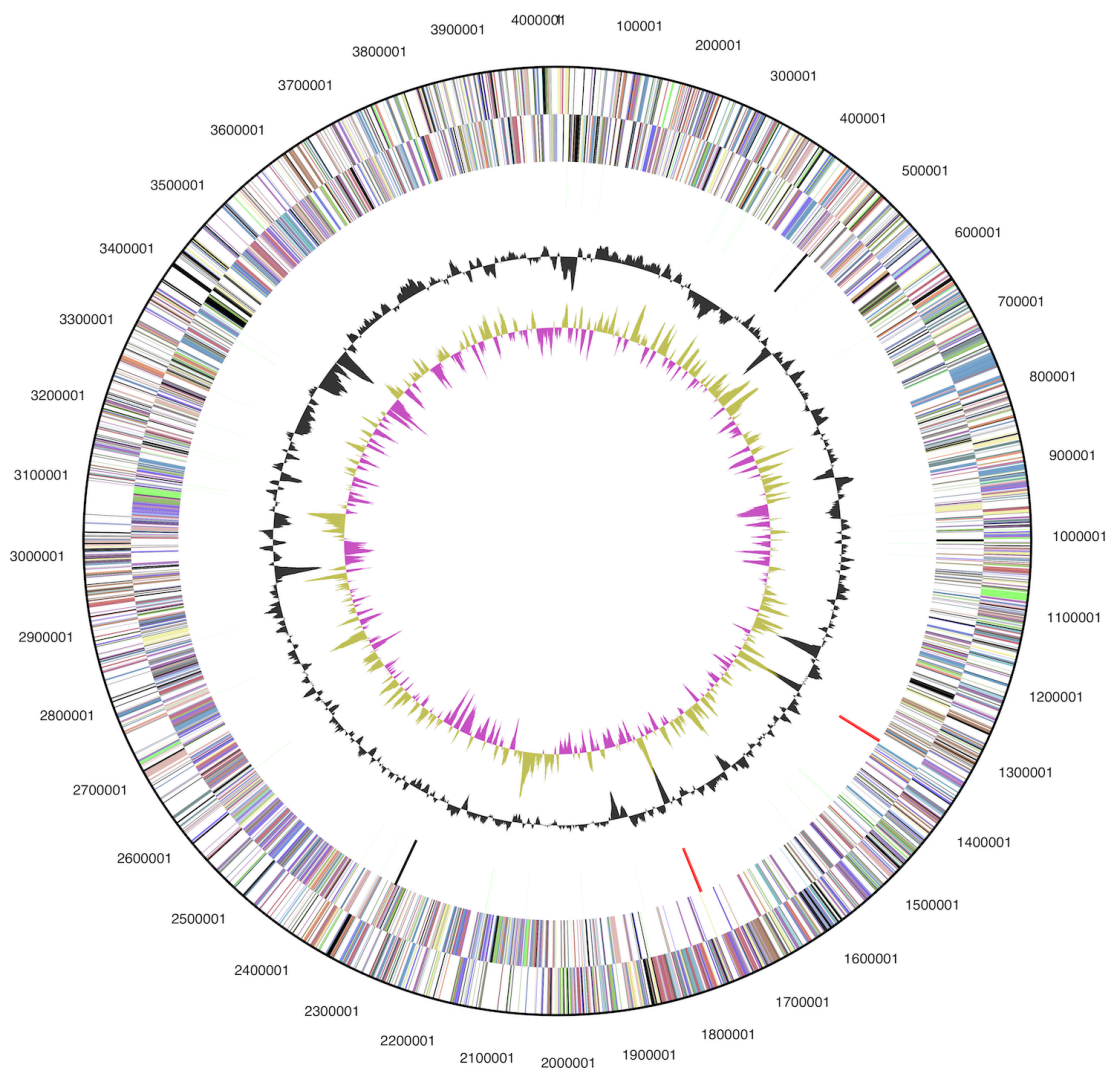
Graphical circular map of the genome. From outside to the center: Genes on forward strand (color by COG categories), Genes on reverse strand (color by COG categories), RNA genes (tRNAs green, rRNAs red, other RNAs black), GC content, GC skew.

The genome consists of a 4,024,382 bp long chromosome with a 70.7% GC content (Table 3 and Figure 3). Of the 3,710 genes predicted, 3,653 were protein-coding genes, and 57 RNAs; ninety pseudogenes were also identified. The majority of the protein-coding genes (71.3%) were assigned with a putative function while the remaining ones were annotated as hypothetical proteins. The distribution of genes into COGs functional categories is presented in Table 4.

**Table 3:** Genome Statistics

**Attribute**	**Value**	**% of Total**
Genome size (bp)	4,024,382	100.00%
DNA coding region (bp)	3,618,708	89.92%
DNA G+C content (bp)	2,845,385	70.70%
Number of replicons	1	
Extrachromosomal elements	0	
Total genes	3,710	100.00%
RNA genes	57	1.54%
rRNA operons	2	
Protein-coding genes	3,653	98.46%
Pseudo genes	90	2.43%
Genes with function prediction	2,645	71.29%
Genes in paralog clusters	360	7.70%
Genes assigned to COGs	2,674	72.08%
Genes assigned Pfam domains	2,871	77.39%
Genes with signal peptides	1,101	29.68%
Genes with transmembrane helices	860	23.18%
CRISPR repeats	0	

**Table 4:** Number of genes associated with the general COG functional categories

**Code**	**value**	**%age**	**Description**
J	160	5.4	Translation, ribosomal structure and biogenesis
A	1	0.0	RNA processing and modification
K	230	7.8	Transcription
L	181	6.1	Replication, recombination and repair
B	2	0.1	Chromatin structure and dynamics
D	38	1.3	Cell cycle control, cell division, chromosome partitioning
Y	0	0.0	Nuclear structure
V	46	1.6	Defense mechanisms
T	131	4.4	Signal transduction mechanisms
M	155	5.2	Cell wall/membrane/envelope biogenesis
N	2	0.1	Cell motility
Z	0	0.0	Cytoskeleton
W	0	0.0	Extracellular structures
U	34	1.2	Intracellular trafficking and secretion, and vesicular transport
O	98	3.3	Posttranslational modification, protein turnover, chaperones
C	223	7.5	Energy production and conversion
G	199	6.7	Carbohydrate transport and metabolism
E	305	10.3	Amino acid transport and metabolism
F	79	2.7	Nucleotide transport and metabolism
H	141	4.8	Coenzyme transport and metabolism
I	151	5.1	Lipid transport and metabolism
P	132	4.5	Inorganic ion transport and metabolism
Q	89	3.0	Secondary metabolites biosynthesis, transport and catabolism
R	358	12.1	General function prediction only
S	212	7.2	Function unknown
-	1,036	27.9	Not in COGs
